# Sustainable chemical technology: Core concepts, key subfields, and assessment approaches

**DOI:** 10.1016/j.bidere.2026.100105

**Published:** 2026-07-25

**Authors:** Máté Hartyányi, Roland Nagy

**Affiliations:** University of Pannonia, Faculty of Engineering, Department of MOL Hydrocarbon- and Coal Processing, Veszprém, Egyetem u. 10., H-8200, Hungary

**Keywords:** Green chemistry, Catalysis, Circular economy, Solvent recovery, Process intensification, Electrification, Sustainability metrics

## Abstract

Sustainable Chemical Technology provides an integrative framework for designing and deploying chemical products and manufacturing routes that reduce life-cycle environmental burdens while maintaining technical performance and economic viability. While green chemistry principles guide safer and more efficient transformations at the molecular level, sustainability outcomes at scale are often dominated by process and system factors, including separations and solvent management, utilities and energy integration, operability, and supply-chain constraints. This Short Communication proposes a concise working definition and a four-level interpretation structure spanning material, process, system, and life-cycle perspectives to support consistent boundary setting and comparison across technologies. Building on this framing, we summarize a compact taxonomy of core subfields feedstock and resources, reaction and process design, solvents and separations, and circularity and waste and outline typical intervention levers and trade-offs within each. Finally, we recommend a minimal assessment bundle that combines fast screening metrics with life-cycle thinking to reduce burden shifting and enable transparent decision-making from early research to industrial adoption.

## 1 Introduction and conceptual foundations

Sustainable chemical technology represents a paradigm shift in how chemical products are designed, manufactured, and used [1]. Green chemistry is defined as the design of chemical products and methods that eliminate or reduce the practice and generation of unsafe and hazardous materials, responding to population growth, depletion of non-renewable fossil resources, and rising atmospheric CO_2_ [1,2]. Unlike traditional approaches that manage pollution after it is produced, sustainable chemistry focuses on preventing hazardous substances at the molecular level and incorporating environmental considerations from the earliest stages of research and development [3,4].

The scope of sustainable chemistry extends across virtually all areas of chemical science, including organic, inorganic, biochemistry, polymer chemistry, toxicology, environmental chemistry, and physical chemistry [5]. The transition toward sustainable chemical technology is driven by converging factors. Environmentally, the chemical industry has contributed to hazardous waste generation, greenhouse gas emissions, and recalcitrant compounds that persist in the environment; green chemistry addresses these concerns through cleaner production technologies, advanced carbon capture concepts, and the development of biodegradable materials aligned with the twelve principles to minimize carbon footprints and resource use [6]. Economically, while initial capital investments may be higher, long-term operational costs can decrease because of reduced waste disposal requirements, lower energy consumption, and improved regulatory compliance, while new markets emerge as consumer demand for eco-friendly products grows [7].

The twelve principles (less hazardous synthesis, atom economy, waste prevention, designing safer chemicals, energy efficiency, safer solvents, reduced derivatives, renewable feedstocks, catalysis, design for degradation, inherently safer chemistry for accident prevention, and real-time analysis) provide the unifying framework for sustainable practice [1,8]. Over the past two decades, major pharmaceutical companies, fine chemical manufacturers, and industrial producers have increasingly implemented green chemistry methodologies, though adoption rates vary by sector and geography [9]. Implementation challenges include technological maturity, economic feasibility, infrastructure investment needs, and policy design effectiveness [10].

**Fig. 1:**
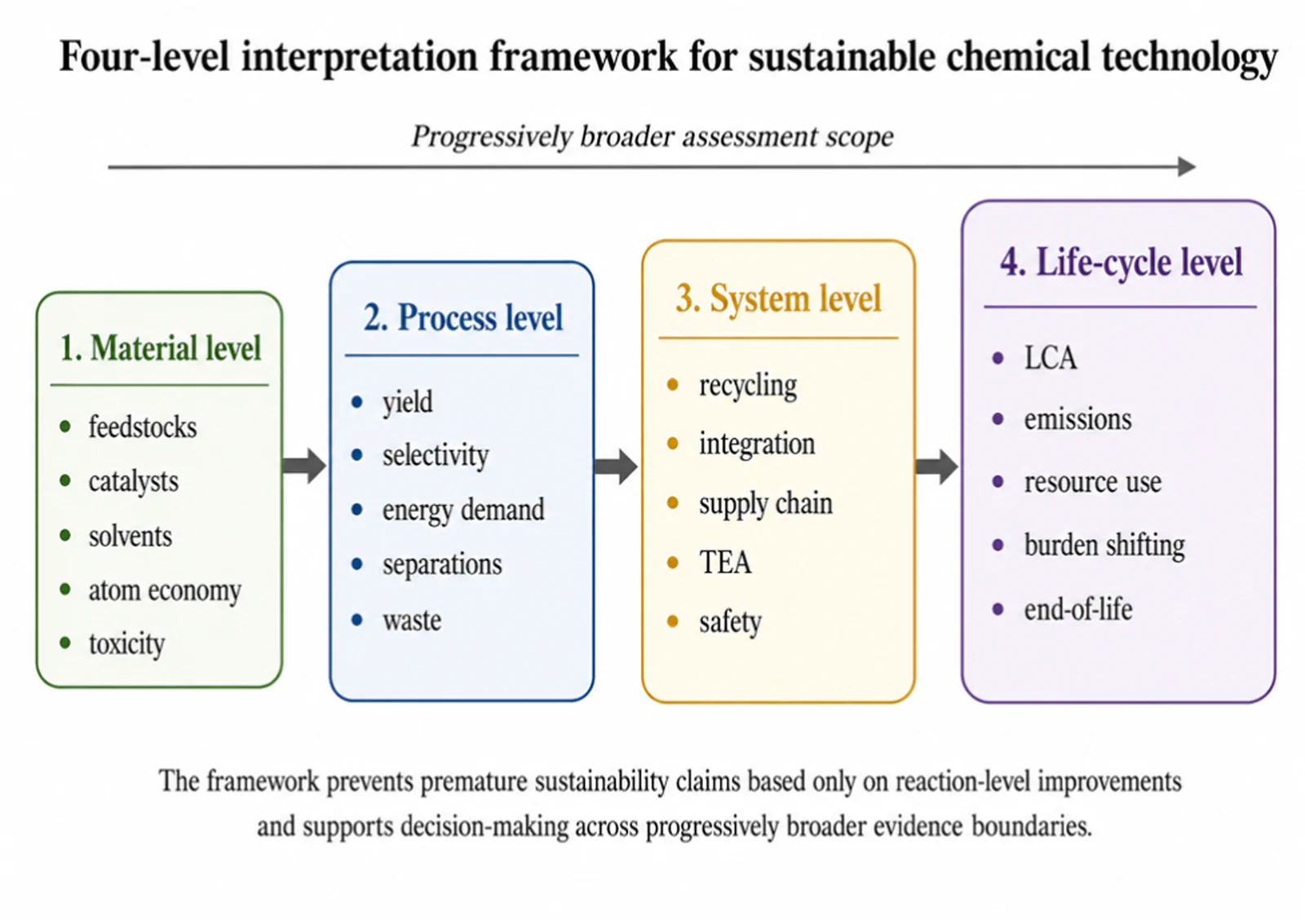
Four-level interpretation framework for sustainable chemical technology.

To organize these diverse sustainability considerations, this Short Communication applies a four-level interpretation framework. The material level focuses on molecules, feedstocks, catalysts, solvents, and intrinsic reaction efficiency; the process level considers reaction conditions, energy demand, separations, waste generation, and recovery; the system level addresses industrial integration, circularity, supply chains, safety, and techno-economic feasibility; and the life-cycle level evaluates broader environmental impacts, burden shifting, emissions, resource use, and end-of-life implications. This framework is summarized in Fig. 1 and used throughout the manuscript to connect green chemistry interventions with broader sustainability assessment.

The framework connects four progressively broader levels of assessment. The material level focuses on molecules, feedstocks, catalysts, solvents, reagents, and intrinsic reaction efficiency. The process level evaluates yield, selectivity, solvent use, separations, energy demand, waste generation, and catalyst recovery. The system level considers industrial integration, circularity, recycling, supply chains, process safety, operability, and techno-economic feasibility. The life-cycle level evaluates environmental impacts across broader boundaries, including raw material acquisition, production, use, emissions, waste treatment, and end-of-life stages. The framework is intended to prevent premature sustainability claims based only on reaction-level improvements and to support more transparent decision-making across material, process, system, and life-cycle evidence.

## 2 Key subfields of sustainable chemical technology

### 2.1 Catalytic technologies

Catalysis is one of the central enabling strategies of sustainable chemical technology because it can increase reaction rates, improve selectivity, reduce the need for stoichiometric reagents, and lower waste formation. In green chemistry, catalytic reagents are generally preferred over stoichiometric reagents because they can reduce material consumption and improve atom economy [11,12]. However, the sustainability of a catalytic process cannot be judged only from conversion or yield. A catalyst that performs well at the molecular level may still provide limited sustainability benefit if it requires scarce elements, toxic ligands, energy-intensive preparation, difficult separation, or frequent replacement. Therefore, catalytic technologies should be assessed across material, process, system, and life-cycle levels, including catalyst origin, activity, lifetime, recovery, regeneration, solvent compatibility, energy demand, and possible end-of-life implications.

Chemocatalysis remains essential in large-scale chemical manufacturing, including hydrogenation, oxidation, C–C coupling, polymerization, biomass upgrading, and CO_2_ utilization. Heterogeneous catalysts are particularly important in industrial practice because they can often be separated more easily from the reaction mixture and integrated into continuous processes [13]. Recent developments increasingly focus on earth-abundant metals, supported metal nanoparticles, single-atom catalysts, organocatalysts, and hybrid catalytic systems [14,15]. These approaches may reduce reliance on rare or toxic metals and improve process compatibility with intensified reactor concepts. Nevertheless, their sustainability advantage depends on turnover number, catalyst stability, metal leaching, deactivation, regeneration requirements, and the environmental burden associated with catalyst synthesis and disposal.

Biocatalysis offers a complementary route to sustainable synthesis by using enzymes or whole-cell systems to perform highly selective transformations under comparatively mild conditions [16,17]. Enzymes can provide high chemo-, regio-, and stereoselectivity, which is especially valuable in the synthesis of chiral pharmaceutical intermediates, fine chemicals, and functional molecules. Advances in directed evolution, protein engineering, enzyme immobilization, and cofactor recycling have expanded the substrate scope and operational robustness of biocatalytic processes [16,17]. A well-established industrial example is the biocatalytic transaminase route to sitagliptin, in which an engineered enzyme replaced a rhodium-catalyzed asymmetric hydrogenation step and enabled a more selective process under milder conditions [18]. From a sustainability perspective, this example illustrates how biocatalysis can reduce the need for precious-metal catalysts and harsh reaction conditions while improving selectivity. At the same time, biocatalytic processes may face limitations related to enzyme cost, substrate inhibition, solvent tolerance, mass transfer, enzyme lifetime, and downstream processing.

Nanocatalysis bridges aspects of homogeneous and heterogeneous catalysis by using nanoscale materials with high surface area, tunable active sites, and enhanced contact between reactants and catalytic centers [15,19]. Metal nanoparticles, metal oxides, carbon-supported catalysts, magnetic nanoparticles, and biomass-derived carbonaceous supports have been investigated as recyclable catalytic systems in green chemistry contexts [15,19]. Single-atom catalysts further extend this concept by maximizing metal atom utilization and offering well-defined active sites, which can improve activity and selectivity while reducing the amount of metal required [14]. However, the apparent sustainability benefits of nanocatalysts should be interpreted cautiously. Catalyst aggregation, metal leaching, difficult recovery, and limited long-term stability may reduce their practical advantage, particularly when repeated reuse or continuous operation is required. Therefore, nanocatalytic systems should be assessed not only by catalytic activity, but also by recyclability, durability, separation efficiency, and material requirements.

Photocatalysis and photoredox catalysis are increasingly important because they enable chemical transformations using light as an energy input. Transition-metal complexes, organic dyes, semiconductor materials, and carbon nitride-based photocatalysts can generate excited states capable of single-electron transfer, energy transfer, or redox transformations under mild conditions [[20], [21], [22], [23]]. In organic synthesis, visible-light photoredox catalysis has become a powerful strategy for generating reactive intermediates under comparatively mild conditions, expanding the toolbox for selective bond formation [[20], [21], [22]]. Semiconductor photocatalysts also provide routes for solar-fuel-related transformations. For example, polymeric carbon nitride has been reported as a metal-free photocatalyst for visible-light-driven hydrogen production from water in the presence of suitable reaction conditions [23]. Nevertheless, the broader implementation of photocatalytic systems requires careful consideration of photon efficiency, catalyst durability, light penetration, reactor design, and process productivity.

Catalytic technologies contribute most effectively to sustainable chemical technology when catalyst performance is connected to process integration and life-cycle consequences. A highly active catalyst at the material level is not automatically sustainable if its preparation, recovery, or disposal creates substantial environmental or economic burdens. Conversely, a moderately active but robust, recyclable, non-toxic, and easily separable catalyst may provide greater system-level benefit. For this reason, catalyst selection should be linked with green metrics, techno-economic considerations, and life-cycle thinking rather than being assessed only through conversion, yield, or selectivity.

### 2.2 Renewable feedstocks and biomass valorization

Replacing fossil-based feedstocks with renewables is a central strategy for sustainable chemistry. Biomass from plants, agricultural waste, and industrial residues is abundant and can support production of fuels and diverse product streams while reducing greenhouse gas emissions and supporting rural economies [24]. Lignocellulosic biomass (cellulose, hemicellulose, lignin) is the most abundant non-food feedstock and can provide platform chemicals to replace fossil building blocks, although yields, harsh processing conditions, costs, and downstream separations remain challenges [25].

Key biomass-derived platform chemicals include furfural, 5-hydroxymethylfurfural (HMF), and levulinic acid [26]. Metal-based catalysts such as Ni, Co, and Fe can be cost-effective for large-scale transformations, while noble metals (Pt, Pd) can provide high activity and selectivity for specific upgrading reactions [27]. Furfural, identified among the most valuable biomass-derived chemicals, can be converted into furan-based epoxide monomers; catalytic ring-opening copolymerization of CO_2_ with furfural-derived epoxides yields polycarbonates and poly (monothiocarbonates) that combine thermal stability with hydrolytic degradability [28]. HMF and related furan derivatives serve as versatile building blocks for fine chemicals and advanced materials, supporting sustainable synthesis [29].

Biomass upgrading includes hydrodeoxygenation, hydrogenation, and reforming, where catalyst selection and reaction condition optimization are critical for desired products and minimal byproduct formation [27]. Sonocatalytic approaches combine ultrasound with catalysis; acoustic cavitation generates localized extreme temperatures and pressures that can enhance reaction rates and enable otherwise challenging transformations [30]. Renewable carbonaceous materials (biochar, hydrochar, activated carbon) derived from diverse biomass sources can act as catalyst supports and show applications in biodiesel production and tar removal [31]. Liquid humins, a byproduct of biomass-to-furanics processing, can be converted into porous, nitrogen-doped carbon materials to improve Pd/C catalyst accessibility and performance, illustrating circular utilization of byproducts [32].

### 2.3 Waste prevention and circular economy integration

A transformative aspect of sustainable chemical technology is its focus on waste reduction and pollution prevention at the source rather than end-of-pipe treatment. The circular economy is an economic model prioritizing resource efficiency by minimizing waste, promoting reuse, and closing material loops; integrating green chemistry with circular economy principles can reshape the industry's relationship with resources and environmental systems [33]. The prevention principle is foundational: processes should be designed to prevent waste formation rather than treating waste after it is produced [3]. Mechanochemical synthesis illustrates prevention through solvent-free processing; bead-mill synthesis of paracetamol via the Beckmann rearrangement achieved higher yields while eliminating solvents, and green metrics and EcoScale scores indicated superior overall performance compared with solution-based routes [34].

Solvent choice remains a critical decision point. Water is an ideal green solvent but is not universally applicable; ionic liquids, supercritical fluids, and bio-based solvents can reduce environmental impact and enable milder reaction conditions [35]. Industrial sustainability depends strongly on solvent recovery and separation technologies; recovery approaches include pressure-swing, batch, and heterogeneous distillation and separation technologies involving ionic liquids, enabling reintegration of solvents into production cycles [35].

Circular economy strategies include business models and valorization pathways. Chemical leasing (payment for service rather than volume) aligns supplier incentives with reduced chemical consumption and waste [36]. Recycling of cotton textile waste can integrate mechanical, chemical, and biological routes within circular economy frameworks [37]. In agriculture, circular economy practices can support climate resilience through residue utilization and integrated resource flows [38]. Fruit and vegetable wastes can be valorized via solid-state fermentation into diverse bio-products and platform chemicals [39]. Circular water management uses physicochemical treatments and advanced wastewater processing to recover water and resources such as biogas and nutrients and to enable selective recovery of high-value metals via membrane and electrochemical methods [40].

### 2.4 Application domains and process intensification

Green chemistry principles are applied across pharmaceuticals, fine chemicals, polymers, agriculture, and environmental remediation. In pharmaceuticals, they can eliminate harmful processes and materials, reduce toxicity, and improve efficiency, including shifts to bio-based feedstocks, while continuous manufacturing can further reduce waste and carbon footprint and increase flexibility [3,41]. In fine and specialty products, the fragrance industry illustrates replacing harsh conditions with renewable feedstocks, efficient catalysts, and benign solvents, and biocatalysis can improve selectivity and lower energy demand [42].

Green synthesis methods including microwave and ultrasound assistance, ball milling, grinding, and photocatalysis can increase reaction rates and selectivity, improving yields while lowering environmental impact and operating costs [43]. Biomass-derived gateway chemicals can also enable sustainable polymers, for example by converting furfural-based epoxides into polycarbonates via catalytic ring-opening copolymerization with CO_2_, producing materials that may be biodegradable under appropriate conditions [28]. Green molecularly imprinted membranes and polymers similarly use safer chemicals, natural polymers, ultrasound assistance, and supercritical CO_2_, supporting applications from pharmaceuticals to materials science [44].

In agriculture, green chemistry can reduce ecological impacts while maintaining productivity through safer alternatives and sustainable practices [45]. Water management and remediation can be supported by circular water economy concepts using chemical engineering and computational simulations to optimize treatment and resource recovery systems [46]. Process intensification further complements green chemistry by miniaturizing reactors and improving parameter control; microreactors enhance heat and mass transfer, and packed-bed microreactors enable efficient heterogeneous catalysis [47].

Microflow chemistry enables continuous manufacturing with improved mixing, residence-time control, productivity, and inherent safety, and electrified microflow systems can couple production with renewable electricity [48]. Ultrasound and microwave assistance can improve yields and selectivity while shortening reaction times compared to conventional heating [49,50]. Electrochemical processing similarly leverages electricity for chemical transformations and can reduce reliance on fossil fuels when powered by renewables [48]. Green power integration includes power storage, water electrolysis for hydrogen, and CO_2_ capture and utilization to support lower-carbon production of chemicals such as ammonia and methanol, although technical bottlenecks and development risks remain [51]. Hybrid photocatalysis–sonochemistry can enhance biomass valorization [30], while bioprocess simulation supports evaluation of alternative scenarios, techno-economic feasibility, and circular economy optimization at scale [52].

The subfields discussed above show that sustainable chemical technology cannot be reduced to a single intervention, such as replacing a solvent, introducing a catalyst, or using renewable feedstocks. Each technology area can provide clear sustainability benefits, but these benefits are usually accompanied by trade-offs related to material availability, process complexity, recovery, scalability, economic feasibility, or life-cycle impacts. To further illustrate the practical relevance of these technology areas, Table 1 includes selected representative examples and industrial or near-industrial cases. These examples are illustrative rather than exhaustive and are included to show how the proposed four-level sustainability assessment framework can be applied to concrete technologies (see Table 2).

**Table 1:** Key sustainable chemical technology areas, expected benefits, and typical trade-offs.

Technology area	Main sustainability lever	Expected benefit	Representative example/industrial case	Typical trade-offs or decision risks
Catalytic technologies	Improved selectivity, lower activation energy, reduced stoichiometric reagents	Lower waste generation, milder conditions, improved atom and material efficiency	Pt_1_/FeO_x_ single-atom catalyst for CO oxidation as an example of maximizing metal atom efficiency [53]	Catalyst cost, deactivation, recovery, leaching, scarce metals, catalyst preparation burden
Biocatalysis	Enzyme- or whole-cell-based selective transformations	High chemo-, regio-, and stereoselectivity; mild operating conditions	Engineered transaminase route to sitagliptin manufacture, replacing a precious-metal-based asymmetric hydrogenation step [18]	Enzyme cost, stability, solvent tolerance, substrate scope, downstream processing
Renewable feedstocks and biomass valorization	Replacement of fossil carbon with renewable carbon sources	Reduced fossil dependence, potential carbon circularity, valorization of residues	Catalytic conversion of biomass-derived carbohydrates to 5-hydroxymethylfurfural or dimethylfuran as platform chemicals/fuels [54,55]	Land use, feedstock variability, pretreatment severity, competition with food or material uses
Safer solvents and solvent recovery	Replacement, reduction, or recycling of solvents	Reduced toxicity, lower solvent losses, improved resource efficiency	CO_2_-switchable hydrophilicity solvents as recyclable solvent systems for extraction and separation [56]	Solvent production burden, separation energy, recyclability, compatibility with reaction and product purification
Process intensification and microflow chemistry	Improved heat and mass transfer, continuous operation, better process control	Higher safety, reduced reactor volume, improved productivity, lower material hold-up	Continuous high-pressure asymmetric hydrogenation with integrated workup and isolation in pharmaceutical process development [57]	Scale-out complexity, equipment cost, clogging, process control requirements
Electrification and renewable energy integration	Substitution of fossil-derived heat or reductants with electricity from low-carbon sources	Potential reduction of process-related emissions and improved coupling with renewable power	Electrochemical CO_2_ reduction on copper electrodes as a representative electrosynthetic route toward carbon-containing products [58]	Electricity source dependence, intermittency, infrastructure needs, energy efficiency
Circular economy and waste valorization	Reuse, recycling, by-product utilization, and closed-loop material flows	Reduced waste disposal, improved resource efficiency, new value from residues	Engineered PET depolymerase for enzymatic depolymerization and recycling of PET bottles [59]	Quality loss, contamination, separation burden, market demand, regulatory constraints
LCA- and TEA-guided technology selection	Integration of environmental and economic evidence	More transparent comparison and reduced risk of burden shifting	Techno-economic and life-cycle assessment of enzymatic PET recycling as an example of combined environmental and economic evaluation [60]	Data intensity, assumptions, allocation choices, uncertainty, scale-up extrapolation

**Table 2:** Selected sustainability metrics and their role in the four-level interpretation framework.

Metric/tool	Main meaning	Main framework level	Key limitation
Atom economy	Fraction of reactant atoms incorporated into the target product	Material/reaction	Does not include yield, solvents, energy, purification, or toxicity
E-factor	Mass of waste per mass of product	Process	Strongly depends on system boundaries and does not directly reflect waste hazard
Carbon efficiency	Fraction of reactant carbon retained in the target product	Material/reaction	Does not capture non-carbon inputs, energy demand, or downstream losses
RMI/PMI	Total input mass required per mass of product	Process	Requires clear definition of included materials and process boundaries
LCA	Environmental impacts across defined life-cycle stages	Life cycle	Data-intensive and sensitive to system boundaries, allocation, and assumptions
TEA	Economic feasibility and scale-up plausibility	System/process	Does not by itself quantify environmental impacts
Burden-shifting analysis	Identification of transferred impacts between stages or categories	System/life cycle	Requires interpretation across multiple metrics rather than one numerical value

This table is not intended as an exhaustive classification. Rather, it summarizes how different sustainable chemical technology interventions can provide benefits at one level while creating new questions at another. The examples illustrate how representative technologies can be interpreted through the proposed four-level framework, while the trade-offs emphasize why sustainability assessment should include both expected advantages and associated decision risks.

## 3 Sustainability assessment and green metrics

Evaluating sustainable chemical technology requires metrics that connect molecular design, process performance, system integration, and life-cycle consequences. No single metric is sufficient to define sustainability. Instead, different indicators are useful at different levels of interpretation. In the four-level framework proposed in this Short Communication, material-level metrics describe the intrinsic efficiency or safety of molecules, feedstocks, catalysts, solvents, and reactions; process-level metrics address material and energy demand during production; system-level assessment considers integration, circularity, recovery, supply chains, and economic feasibility; and life-cycle-level assessment evaluates environmental impacts across broader product or process boundaries.

Atom economy is a material- and reaction-level metric that expresses the fraction of reactant atoms incorporated into the desired product. It is useful for comparing synthetic routes before experimental optimization, but it does not include yield, solvent use, purification, energy demand, or the toxicity of reagents and by-products [12,61]. The E-factor, defined as the mass of waste generated per mass of product, shifts attention from yield alone to waste formation and is particularly useful for comparing process alternatives when material balances are available [12,62]. Reaction mass intensity (RMI) and process mass intensity (PMI) express the amount of input material required to produce a given amount of product. These metrics are valuable because they include material demand more directly and can highlight the contribution of solvents, work-up, and auxiliary materials [63]. Carbon efficiency is useful when renewable carbon utilization or carbon retention is central to the process, but it should not be interpreted independently of total material demand, energy use, or downstream processing.

LCA and TEA provide broader evaluation layers. LCA can assess environmental impacts across the product or process life cycle, including raw material acquisition, production, use, and end-of-life stages, depending on the selected system boundary [64]. TEA complements this by examining whether a technically promising route is economically plausible at relevant scale, considering factors such as feedstock cost, utilities, capital investment, operating costs, and product value [65]. These tools are especially important because improvements in one metric can create burden shifting. For example, a route with high atom economy may still require hazardous solvents or energy-intensive separations, while a route with low direct waste generation may transfer environmental burden to catalyst production, feedstock supply, or end-of-life treatment.

A widely recognized example is the redesign of ibuprofen manufacture. The original Boots route was a six-step synthesis, whereas the later BHC/Celanese process introduced a shorter and more catalytic three-step route [66,67]. If this example is evaluated only at the material or reaction level, the decision appears straightforward: the shorter route is preferable because it improves atom utilization and reduces stoichiometric waste formation. The four-level framework changes this interpretation by turning a simple “greener route” statement into a staged decision. At the material level, the question is whether the reaction pathway uses atoms, reagents, and catalysts more efficiently. At the process level, the decision must also consider step count, yield, solvent use, separations, waste streams, and catalyst recovery. At the system level, the relevant question becomes whether the route can be integrated into safe and reliable industrial operation, including recycling, acid handling, process safety, and supply-chain constraints. At the life-cycle level, the final sustainability claim requires broader evidence on raw material supply, utilities, emissions, waste treatment, and end-of-life implications. Thus, the proposed framework changes the decision-making logic from selecting the route with the best reaction-level metric to identifying which route remains advantageous when material, process, system, and life-cycle evidence are considered together.

For early-stage research, the most practical approach is therefore not to search for a single “best” metric, but to combine a small bundle of complementary indicators. Atom economy and carbon efficiency can screen the theoretical efficiency of a reaction route at the material level; E-factor, RMI, and PMI can indicate material intensity and waste generation at the process level; TEA can test system-level feasibility; and LCA can evaluate whether the apparent improvement remains meaningful across broader life-cycle boundaries. This combined interpretation supports the four-level framework proposed here: reaction-level improvements are necessary but not sufficient, and robust sustainability claims require connection between material, process, system, and life-cycle evidence.

## 4 Challenges and future perspectives

Substantial challenges remain in scaling sustainable chemical technologies to industrial levels. Catalyst deactivation can limit stability under harsh operating conditions, requiring regeneration or replacement that reduces sustainability advantages, and lack of real-world process data for emerging technologies (including chemical recycling) complicates comprehensive risk assessment [68]. Economic feasibility and cost competitiveness remain barriers: life cycle assessments can show environmental benefits, but capital costs for novel technologies can be higher than for conventional processes, motivating continued innovation in catalysis, process design, and manufacturing [10].

Supportive policy frameworks and regulatory mechanisms are needed to incentivize green chemistry adoption while discouraging harmful practices; fragmented policies can create inconsistent requirements, and cohesive long-term incentives are identified as important for accelerating industrial transformation [69]. Education and workforce development are similarly critical: green chemistry must be integrated across education levels and professional development to enable chemists to design and implement sustainable processes, including integration of environmental and economic considerations across chemistry curricula [70].

The sustainability of emerging technologies requires careful evaluation to avoid unintended consequences; nanoparticle toxicity and broader environmental impacts must be studied before widespread nano-catalyst deployment [68]. Bioeconomy development must balance renewable resource benefits with sustainable supply chains, competition with food production, and food security considerations [71]. Future opportunities include integration of artificial intelligence and machine learning with catalyst design to accelerate discovery of novel catalytic systems optimized for sustainability metrics [27]. Direct utilization of renewable energy sources via photocatalysis and electrosynthesis could transform the energy footprint of manufacturing; hydrogen and carbon dioxide, when produced or captured sustainably, can act as building blocks for a sustainable interface between energy and chemistry [72].

## 5 Summary and conclusions

Sustainable chemical technology, grounded in green chemistry principles, provides a framework for developing environmentally benign and economically viable chemical processes across application domains [1,8]. Progress in catalytic systems, renewable feedstocks, circular economy integration, and process intensification illustrates that sustainability and industrial competitiveness can reinforce each other. Realizing full potential requires continued advances in catalyst efficiency and selectivity, improved economic competitiveness of green technologies, stronger policy support, and comprehensive education and workforce development [10,72].

## Data availability

No new data were generated or analyzed in this study.

## CRediT author statement

Máté Hartyányi: Writing, Conceptualization.

Roland Nagy: Conceptualization, Supervision.

## Declaration of generative AI and AI-assisted technologies in the manuscript preparation process

During the preparation of this work the authors used ChatGPT in order to language editing and text structuring. After using this tool/service, the author(s) reviewed and edited the content as needed and take(s) full responsibility for the content of the published article.

## Funding

This research did not receive any specific grant from funding agencies in the public commercial or not for profit sectors.

## Declaration of competing interest

The authors declare that they have no known competing financial interests or personal relationships that could have appeared to influence the work reported in this paper.
